# A multi-item Physician Global Assessment scale to assess psoriasis disease severity: validation based on four phase III tofacitinib studies

**DOI:** 10.1186/s12895-019-0088-2

**Published:** 2019-06-07

**Authors:** Kristina Callis Duffin, Andrew G. Bushmakin, Joseph C. Cappelleri, Lotus Mallbris, Carla Mamolo

**Affiliations:** 1 Department of Dermatology, University of Utah School of Medicine, Salt Lake City, UT USA; 2 Pfizer Inc, 445 Eastern Point Road, Groton, CT 06340 USA; 3 Pfizer Inc, Collegeville, PA USA

**Keywords:** Psoriasis, Physician Global Assessment, Tofacitinib

## Abstract

**Background:**

Several different Physician Global Assessment (PGA) versions have been used in clinical studies as a co-primary end point to evaluate psoriasis severity. Tofacitinib is an oral Janus kinase inhibitor. We performed an analysis of the PGA using data from studies of tofacitinib in moderate to severe chronic plaque psoriasis.

**Methods:**

Data from 3641 patients with moderate to severe chronic plaque psoriasis, enrolled in one of four phase III tofacitinib studies (OPT Pivotal 1 and 2, OPT Compare and OPT Retreatment), were used to evaluate a three-item PGA scale.

**Results:**

Confirmatory Factor Analyses showed that equal weighting of the three items (erythema, induration and scaling) was appropriate. The PGA demonstrated acceptable test–retest reliability (Intraclass Correlation Coefficient, 0.7) and internal consistency (Cronbach’s Coefficient Alpha ≥ 0.9 at primary time points). The Clinically Important Difference was estimated as 0.55 (95% confidence interval: 0.546–0.563). Known-group validity was shown by demonstrating that PGA scores could discriminate between different degrees of disease severity. The PGA was significantly correlated with other clinical end points in the studies (Psoriasis Area and Severity Index, r = 0.75–0.79; Dermatology Life Quality Index, r = 0.44–0.57; Patient Global Assessment, r = 0.66–0.72).

**Conclusions:**

Consistent with previous findings from a phase II study, these results indicate that this PGA is a valid, reliable instrument for evaluating disease severity in clinical studies of psoriasis.

**Electronic supplementary material:**

The online version of this article (10.1186/s12895-019-0088-2) contains supplementary material, which is available to authorized users.

## Background

The Physician Global Assessment (PGA) is a frequently used co-primary end point in psoriasis clinical trials. The PGA was introduced in 1998 by a US Food and Drug Administration panel as the preferred tool to assess and record the severity of disease in clinical studies, and typically rates a patient’s disease from ‘clear’ to ‘severe’ or ‘very severe’ [1, 2]. The PGA provides a simple subjective measurement of the clinical signs of psoriasis, typically erythema, induration and scaling, across the whole body [3]; however, there is currently no recognised standard definition of the PGA. Multiple versions are currently in use, all varying in terms of the number and description of the items (or symptoms) assessed, and the point values used to rate each item, with the most common PGA versions using five- to six-point scales [3].

Tofacitinib is an oral Janus kinase inhibitor. A three-item version of the PGA was developed for use in psoriasis clinical trials of tofacitinib [4]. This PGA scale asks physicians to rate erythema, induration and scaling, individually, on a five-point scale (from 0 = no symptom to 4 = severe). The total score is the mean of the three item scores, each having an equal weighting [4]. PGA scores based on a multi-item scale are less likely to be affected by random variations than scores based on single-item scales [5], and previous findings from a phase II clinical trial demonstrated the reliability and validity of the three-item PGA for the assessment of psoriasis severity [4].

The objective of the present study was to perform a more intensive and rigorous analysis of the PGA using data from four phase III studies of tofacitinib in moderate to severe chronic plaque psoriasis. This comprised validation of the PGA scoring algorithm using Confirmatory Factor Analysis (CFA), assessment of reliability and internal consistency of PGA measurements; definition of the Clinically Important Difference (CID); evaluation of the ability of the PGA to discriminate between different degrees of disease severity; and correlation of the PGA with other clinical outcome measures.

## Methods

### Studies and patients

Data from four phase III clinical studies of tofacitinib in patients with psoriasis (OPT Pivotal 1 [6], OPT Pivotal 2 [6], OPT Compare [7] and OPT Retreatment [8]) were used. Full details of the studies have been described elsewhere [6–8]. Briefly, OPT Pivotal 1 and 2 (ClinicalTrials.gov identifiers: NCT01276639 and NCT01309737) were similar 52-week phase III studies investigating tofacitinib 5 mg twice daily (BID) and 10 mg BID versus placebo [6].

The primary assessment time point was week 16. OPT Compare (NCT01241591) compared tofacitinib 5 mg BID or 10 mg BID with etanercept 50 mg twice weekly and placebo; the primary end point was assessed at week 12 [7]. OPT Retreatment (NCT01186744) was a treat–withdrawal–retreatment study of tofacitinib 5 mg BID and 10 mg BID versus placebo for up to 56 weeks [8].

Patients in all four studies were ≥ 18 years of age, diagnosed with chronic (≥ 12 months) plaque psoriasis, had a Psoriasis Area and Severity Index (PASI) score of ≥ 12, a PGA of moderate or severe, and psoriasis that involved at least 10% of their body surface area [6–8]. All patients were candidates for systemic or phototherapy [6–8]; in OPT Compare, patients had to have failed to respond to, had a contraindication to, or been intolerant to, at least one conventional systemic therapy (including ultraviolet therapy) approved for plaque psoriasis treatment [7]. For this analysis, data from the primary efficacy assessment at week 24 (continuous treatment) were used [8].

### Assessments

The co-primary end points in all four studies were the PGA and PASI. The PGA assessed three items (erythema, induration and scaling) on a scale from 0 = clear to 4 = severe. Items were rated separately across all psoriatic lesions and scored from 0 to 4 based on morphological descriptors. Severity rating scores for each item were summed and the mean taken; the mean was rounded to the nearest integer to determine the PGA score.

The PASI is a summary score evaluating the three signs of lesion severity (erythema, induration and scaling) according to a five-category scale for each of four anatomical regions of the body (head/neck, upper extremities, trunk and lower extremities) [1]. Higher PASI scores represent increasing psoriasis severity.

Where justified, evaluations were conducted using pooled data from all four studies to streamline and generalise results, as well as interpretation.

### Confirmatory Factor Analysis

**Fig. 1:**
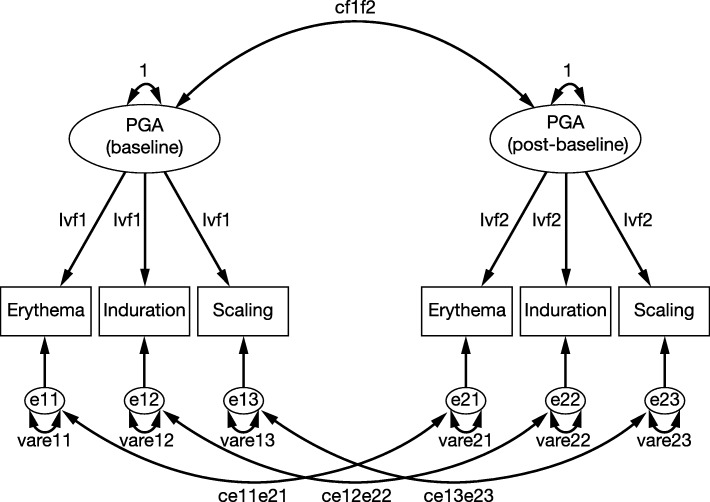
Pathways in the measurement model used in the Confirmatory Factor Analysis in phase III studies. Equal weighting of each item (erythema, induration, scaling) is assumed. Ovals represent unobserved (latent) factors (i.e., PGA at baseline and post-baseline); rectangles represent observed items (e.g., erythema, induration, scaling). Loadings from PGA to each item are symbolised by ‘lvf1’ (baseline) and ‘lvf2’ (post-baseline). Residual or error terms represent all factors influencing variability in an item other than the latent factor that precedes and predicts that item. Error terms begin with ‘e’ and end with two digits indicating the item measured (e.g., ‘e11’ = error term for erythema and corresponding variances begin with ‘var’). Covariances between pairs of error terms (latent factors) begin with ‘c’ (e.g., ‘ce11e21’ = covariance of the error term between erythema at baseline and at post-baseline). *PGA* Physician Global Assessment

CFA was used to test the fit of the PGA measurement model. Bentler’s Comparative Fit Index (CFI) was used as a measure of fit of the model with the data. Acceptable fit was defined as a CFI > 0.9; in addition, path coefficients had to be statistically significant and standardised path coefficients had to be > 0.4 [9–11]. The analysis was performed both with path coefficients constrained to be equal (reflecting the PGA scoring algorithm having equal weighting for each item) and with such constraints removed (Fig. 1).

### Test–retest reliability

The PGA was performed for each patient on at least two different occasions under a relatively stable set of conditions before treatment intervention, allowing assessment of instrument stability and replicability. Test–retest reliability was evaluated by estimating a Dermatology Life Quality Index (DLQI) based on pooled PGA data. All available pre-treatment data (screening and baseline) were used. In addition, sensitivity analyses were performed using data from each study separately. An Intraclass Correlation Coefficient ≥ 0.70 was considered satisfactory [12, 13].

### Internal consistency reliability

Internal consistency reliability was assessed by calculating Cronbach’s Coefficient Alpha and corrected item-to-total correlations (correlation of an item with the total score excluding that item) at baseline and the primary assessment time points. A Cronbach’s Coefficient Alpha of ≥ 0.7 was considered acceptable [12, 13].

### Clinically Important Difference

CID for the PGA was defined by using a repeated measures model [13] to estimate the relationship between PGA and Patient Global Assessment (PtGA) scores. A linear relationship was imposed where PGA was the outcome and PtGA was a continuous anchor. This model incorporated all available data across all time points for all studies from baseline to the primary assessment time point. The mean difference on the PGA for a one-category difference on the PtGA was taken as the estimated CID.

A sensitivity analysis was performed with PtGA as a categorical anchor. Doing so does not impose any functional relationship between an outcome and an anchor.

### Known-group validity

For known-group validity [13], a repeated measures model was applied to assess the relationship between PGA and PASI. Pooled data for all four studies were analysed. PGA score was used as the outcome and PASI score as a categorical anchor (and hence no functional relationship was imposed). This model included all available data at all time points for all studies from baseline to the primary assessment time point. Seven categories were created: category 0 (observations when patient PASI score was exactly 0, i.e., ‘healthy’) to category 6 (observations when patient PASI score was in the range of 40 to 72), indicative of severe disease.

### Convergent and divergent validity

Convergent and divergent validity [13] were assessed by determining the correlation of the PGA with the PtGA, PASI and DLQI. Evidence for convergent validity was based a priori on a Pearson correlation coefficient of ≥ 0.40, consistent with a meaningful correlation [14]. Regarding divergent validity, evidence was based on a Pearson correlation coefficient of ≤ 0.30, consistent with a less than medium association [15]. Insufficient evidence to dismiss either convergent validity or divergent validity was based on correlations between 0.30 and 0.40 [12].

Additional scales included a PtGA of disease severity and DLQI. The PtGA was a five-point scale using the same category labels as the PGA and reflects the patient’s overall impression of their disease severity at a given time point. The DLQI is a validated general dermatology questionnaire, using 10 items to assess health-related quality of life [16, 17].

### Ethics approval and consent to participate

All clinical studies were conducted in compliance with the ethical principles originating in, or derived from, the Declaration of Helsinki, and with the International Council for Harmonisation Good Clinical Practice Guidelines. All documentation was reviewed by the institutional review board and/or independent ethics committee at each of the investigational centres (Additional file 1). All patients provided written informed consent.

## Results

This analysis included data for 3641 patients with moderate to severe chronic plaque psoriasis who had participated in one of four phase III clinical studies of tofacitinib in patients with psoriasis (OPT Pivotal 1 [NCT01276639] [6], OPT Pivotal 2 [NCT01309737] [6], OPT Compare [NCT01241591] [7] and OPT Retreatment [NCT01186744] [8]). Baseline characteristics were broadly similar across all four studies. Median ages were 44–46 years (range 18–83), 68–71% of patients were male, 81–92% were white and median weights were 83–91 kg (range 36–219 kg). PASI scores at baseline were similar between studies, ranging from a median of 18.4 in OPT Retreatment to 20.5 in OPT Compare. Baseline PGA scores defined 82–90% of patients as moderate and 10–17% as severe. PtGA scores were 29–35% moderate and 61–67% severe.

### Confirmatory Factor Analysis

CFA is a hypothesis-confirming technique to test whether data support a hypothesised measurement model [13]. Here, the CFA model assumed that the three items (erythema, induration and scaling) are equally weighted in PGA. Results of the CFA with path coefficients associated with the three items constrained to be equal (representing the current PGA scoring algorithm), and CFA without these constraints demonstrated excellent fit, indicated by CFI values > 0.98 and standardised path coefficients all above the threshold of 0.4 (Table 1).

**Table 1:** PGA Confirmatory Factor Analysis

Study	CFI^a^ (constrained paths)	CFI^a^ (unconstrained paths)		Difference between path coefficients (constrained vs unconstrained), %		
				Erythema	Induration	Scaling
OPT Pivotal 1	0.995	0.996	Baseline	−18.1	16.7	4.2
			Week 16	0.5	1.9	−1.6
OPT Pivotal 2	0.983	0.995	Baseline	−9.5	18.7^*^	−5.0
			Week 16	−5.5^*^	5.0	−1.8
OPT Compare	0.988	0.997	Baseline	−16.8^*^	16.6^*^	0.6
			Week 12	−3.2	5.5	−3.9
OPT Retreatment	0.981	0.994	Baseline	−34.3^*^	38.9^*^	0.8
			Week 24	−6.0	2.8^*^	0.9

^*^p < 0.05 vs zero

^a^Bentler’s Comparative Fit Index (CFI)

*PGA* Physician Global Assessment

Furthermore, when comparing the ‘relaxed constraints’ paths and the estimated common loadings, 17 out of 24 paths did not differ significantly from each other (Table 1) and those that did occurred at baseline (when there was less assurance given the limited range of the scores from a relatively homogeneous, pre-treatment sample) or were not sizable enough to be meaningful at the primary time assessments. In general, these results support equal weighting of erythema, induration and scaling in the PGA scoring algorithm.

### Test–retest reliability

This technique evaluated consistency of PGA measurements between screening and baseline visits, when no change in terms of disease severity was anticipated. Intraclass Correlation Coefficient values between 0.7 and 0.9 are considered to represent acceptable reliability, while values > 0.9 are usually interpreted as representing excellent reliability [12, 13]. The estimated Intraclass Correlation Coefficient of the PGA for the pooled data was 0.70, thereby suggesting acceptable test–retest reliability that indicates consistency of the PGA scoring over a stable period. Intraclass Correlation Coefficient estimations calculated separately for each study were acceptable for two studies (OPT Pivotal 2, 0.79; OPT Compare, 0.70) and less so for the other two studies (OPT Pivotal 1, 0.60; OPT Retreatment, 0.65).

### Internal consistency reliability

Internal consistency reliability was used to determine if scoring of erythema, induration and scaling were consistent with each other. The PGA scale rendered excellent internal consistency reliability, as indicated by a Cronbach’s Coefficient Alpha ≥ 0.9 in all calculations at the primary assessment time points (OPT Pivotal 1, 0.94; OPT Pivotal 2, 0.95; OPT Compare, 0.92; OPT Retreatment, 0.94). The Cronbach’s Coefficient Alpha values observed at baseline (OPT Pivotal 1, 0.50; OPT Pivotal 2, 0.63; OPT Compare, 0.63; OPT Retreatment, 0.51) were smaller as a result of the pre-selection of relatively homogeneous subjects at baseline (due to the inclusion and exclusion criteria for the clinical trials) whose responses were generally within a restricted range, resulting in small pairwise correlations as expected. These findings indicated that the three item scores were consistent with each other and, therefore, showed concordance with each other.

### Clinically Important Difference

**Fig. 2:**
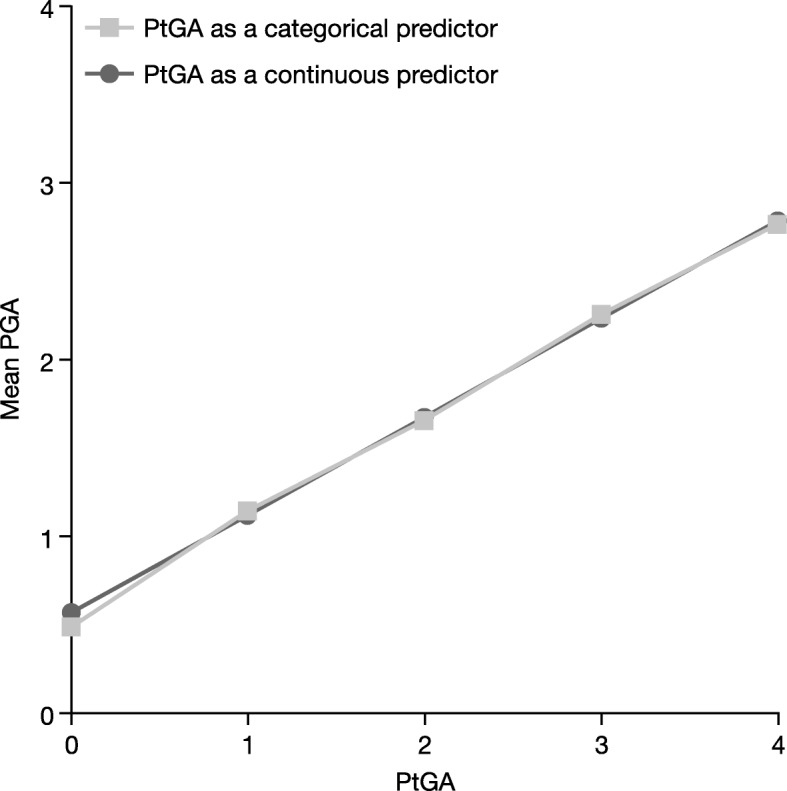
Relationship between PGA score and PtGA category. *PGA* Physician Global Assessment, *PtGA* Patient Global Assessment

The CID is the magnitude of change in PGA that is discernible, clinically and meaningfully, as a difference in disease severity to a patient. With PtGA as a continuous anchor, CID for the PGA score was 0.55 (95% confidence interval: 0.546–0.563), based on pooled data from all four studies, which corresponded to a one-category difference in the PtGA. Figure 2 shows a plot of the PtGA against the PGA when used as either a continuous or categorical predictor. The functional relationship between PGA and PtGA is clearly linear and positive. Results on CID from individual studies were similar (0.53 for OPT Pivotal 1 and 2, to 0.62 for OPT Retreatment), which justified the pooling of the results.

### Known-group validity

**Fig. 3:**
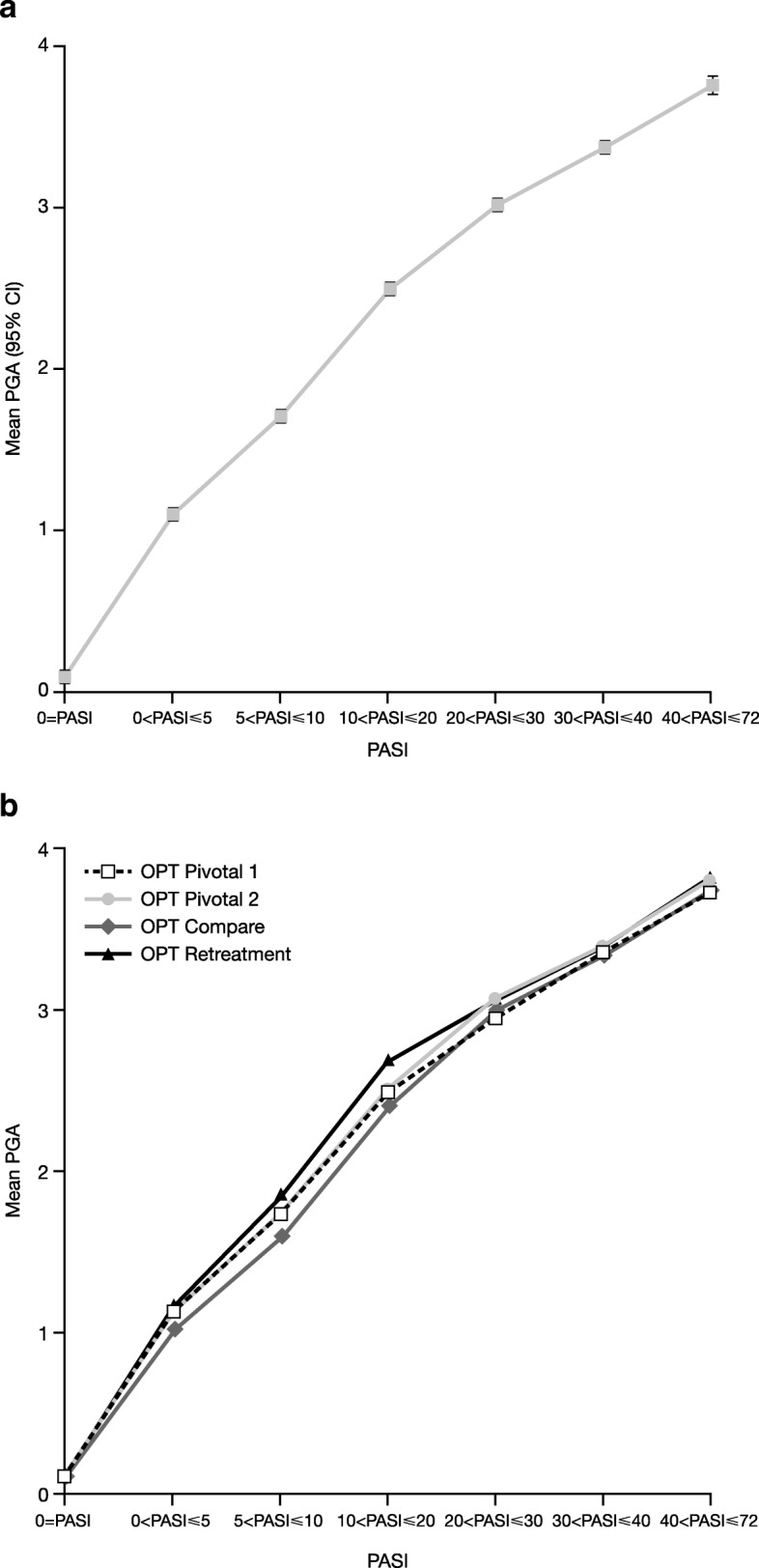
Relationship between PASI score and PGA score. (**a**) Pooled data; (**b**) Individual studies. *CI* confidence interval, *PASI* Psoriasis Area and Severity Index, *PGA* Physician Global Assessment

Known-group validity analysis was used to assess whether PGA could discriminate between different degrees of psoriasis severity, using PASI as a measure of disease severity. A clear positive relationship between PGA and PASI scores was observed (Fig. 3a). The differences in the PGA scores between the ‘clear’ group (PASI score of 0) and the other groups (PASI score > 0) were statistically significant (Table 2), and increased as psoriasis became more severe (i.e., with larger PASI scores). Differences were all greater than the CID, indicating that they were also clinically relevant.

**Table 2:** Known-group validity of the PGA

Category definition based on the PASI score	Mean difference in PGA scores between group with PASI score = 0 and indicated PASI score group^*^	95% CI	
		Lower	Higher
0 < PASI ≤ 5	−1.01	−1.04	−0.98
5 < PASI ≤ 10	−1.61	−1.65	−1.58
10 < PASI ≤ 20	−2.41	−2.44	−2.37
20 < PASI ≤ 30	−2.92	−2.96	−2.89
30 < PASI ≤ 40	−3.27	−3.31	−3.23
40 < PASI ≤ 72	−3.67	−3.73	−3.61

^*^All *p*-values ≤0.0001 vs group with PASI score = 0

PGA score was outcome and PASI score was categorical anchor, with no functional relationship imposed

*CI* confidence interval, *PASI* Psoriasis Area and Severity Index, *PGA* Physician Global Assessment

Analysing the data for each study separately confirmed the very close relationship between PGA and PASI (Fig. 3b), which was stable and replicable across the four phase III studies (Fig. 3b). A robust monotonic functional relationship between PASI and PGA was observed, and showed that the PGA scale can separate between groups that are known to be different; in this case, a clear group (no psoriasis) and groups with psoriasis.

### Convergent and divergent validity

This analysis was performed to assess whether PGA correlated sufficiently with other clinical end points used in the phase III studies; namely: PASI, PtGA and DLQI. Pearson correlation coefficients between the PGA and each of PASI, PtGA and DLQI at primary assessment time point were > 0.4 (Table 3), indicating, as expected, significant and substantial correlations of PGA with these clinical end points. The correlations were not extremely large (above 0.80), indicating that PGA captures some different information compared with PASI and DLQI. In addition, it should be noted that at baseline the correlations diverged and were smaller (Pearson correlation coefficients: PGA vs PASI, 0.28–0.39; PGA vs PtGA, 0.09–0.10; PGA vs DLQI, 0.09–0.14), as expected, stemming from the pre-selection of homogeneous subjects with a limited range of responses.

**Table 3:** Pearson correlation coefficients at the primary assessment time point

Study	Pearson correlation coefficients (Prob > |r| under H0: Rho = 0) Number of observations		
	PGA vs PASI	PGA vs PtGA	PGA vs DLQI
OPT Pivotal 1	0.77 (*p* < 0.0001) *798*	0.70 (*p* < 0.0001) *790*	0.57 (*p* < 0.0001) *789*
OPT Pivotal 2	0.79 (*p* < 0.0001) *851*	0.72 (*p* < 0.0001) *841*	0.53 (*p* < 0.0001) *843*
OPT Compare	0.79 (*p* < 0.0001) *1027*	0.66 (*p* < 0.0001) *1011*	0.53 (*p* < 0.0001) *1008*
OPT Retreatment	0.75 (*p* < 0.0001) *555*	0.70 (*p* < 0.0001) *549*	0.44 (*p* < 0.0001) *549*

*DLQI* Dermatology Life Quality Index, *PASI* Psoriasis Area and Severity Index, *PGA* Physician Global Assessment, *PtGA* Patient Global Assessment

Italic values represent the number of observations

## Discussion

PGA scales are commonly used as a co-primary or secondary end point to assess treatment efficacy in psoriasis clinical trials. However, multiple versions of the PGA currently exist and no standard definition has yet been established. For example, a previous study reported the validation of three measures of physician-reportedpsoriasis severity, using data from 445 patients participating in a single phase III clinical trial. The static Physician’s Global Assessment (sPGA), one of the three measures, is similar to the PGA validated in the current study, in that it assesses erythema, induration and scaling; however, while the PGA reported here uses a scale from 0 = clear to 4 = severe, in the sPGA described by Simpson et al., a scale from 0 to 5 is used. In both cases, each component is equally weighted and the score derived as the mean of the three components [18]. This lack of consensus partly explains why, despite their simplicity, PGA scales are not routinely used by practising clinicians. Therefore, it is important to assess the validity and reliability of each PGA scale for evaluating psoriasis disease severity.

This analysis provides further validation of the three-item PGA tool used in the tofacitinib clinical development programme in a large patient cohort using pooled data from four phase III studies, following preliminary validation based on data from a single phase II study [4]. We confirmed the properties and scoring algorithm of the tofacitinib PGA scale. The data support equal weighting of each of the three PGA items, the three PGA items were consistent with each other and the PGA was reliable over time.

We also showed that PGA and PASI follow a similar pattern with worsening disease, PGA scores could discriminate degrees of psoriasis severity based on PASI and differences in PGA between PASI categories were clinically relevant. Together, these data provide a robust validation of the three-item PGA tool for the assessment of psoriasis.

Finally, we established the CID for this PGA scale as equal to 0.55 points. When evaluating CIDs, it is most desirable to base them on a patient-reported measure, such as the PtGA used here. Although there were differences between PGA and PtGA, they were likely due to differences in patient and physician perspectives of psoriasis severity [19]. In addition, a close to linear relationship was demonstrated between PGA and PtGA when PtGA was used as a categorical anchor, supporting the main model which imposed a linear relationship between PGA and PtGA. In sensitivity analyses using data from each of the studies separately, both for the CID and other analyses, the results for each phase III study were similar and consistent with the previously published phase II results [4], demonstrating the stability and replicability of the PGA across five studies.

This finding is also consistent with a previously published systematic review comparing PASI with various PGA scales reported in the literature, which indicated that PGA and PASI correlate well but concluded that standardisation and validation of PGA was required [20].

Although the evidence clearly shows the favourable measurement properties of this PGA scale, there are potential limitations to consider when interpreting the results of this analysis. For example, the data used were taken from patients with relatively severe disease at baseline and who were being treated within the clinical trial environment; therefore, caution should be used when generalising these findings to the wider psoriasis population. Similarly, as patients were recruited to the studies according to set criteria, this resulted in a relatively homogeneous population at baseline, reflected by the smaller Cronbach’s Coefficient Alpha observed at baseline. Following treatment, there was a greater degree of heterogeneity in the sample and the wider variation scores allow for a more accurate assessment. However, we have not evaluated the sensitivity of PGA to beneficial treatment in this current investigation as this was found and reported elsewhere [6–8].

In addition, a strong correlation with PASI was observed, despite the fact that, unlike the PASI, the PGA does not take into account the amount of body surface area affected by psoriasis. The PGA also showed a moderately strong correlation with PtGA and DLQI. Clinical measures of disease severity such as the PGA and PASI were designed to assess clinical features of psoriasis (erythema, induration and scaling), and not necessarily to evaluate the full impact of symptoms on patients and their quality of life, thus clearly justifying the use of patient-reported as well as physician-reported outcome measures in clinical trials. In general, our findings are consistent with other evidence in the literature demonstrating a correlation between improved PGA scores and increases in patient-reported measures of health-related quality of life in patients with moderate to severe psoriasis [21, 22].

The PGA was designed to be a relatively more simple assessment of erythema, induration and scaling than the PASI, with its more complex scoring algorithm. However, none of the PGA’s items independently and wholly represents the disease and, equally importantly, the PGA does not include quantification of the area of involvement, a critical element to be considered when assessing psoriasis severity, nor does the PGA consider the locations of individual lesions. Furthermore, the precision of PGA scores highly relies on the evaluators strictly adhering to a standard definition of the PGA. PGA scales are therefore useful to assess clinical study end points of disease severity, but are no substitute for thorough clinical assessment in routine practice. No matter how well validated the PGA is, its assessment is limited to what it is intended to measure and, therefore, its breadth must be acknowledged.

Nevertheless, the PGA remains a useful measure in clinical trials of psoriasis, particularly since it is more simple and has more intuitive severity categories, scoring and interpretation than the PASI. As this work attests, the PGA scale used in tofacitinib studies of psoriasis, with its favourable properties, and the relevant scoring algorithm (equal weighting of erythema, induration and scaling) is supported robustly by all available empirical evidence.

## Conclusions

Consistent with previous findings from a phase II study, these results provide needed confirmation and reassurance for a clinician on the suitability of the three-item PGA scale as a reliable, valid measure to obtain a global assessment on the severity of plaque psoriasis in clinical studies.

## Additional file

Additional file 1: List of independent ethics committees or institutional review boards. (DOCX 47 kb)

## Data Availability

Upon request, and subject to certain criteria, conditions and exceptions (see https://www.pfizer.com/science/clinical-trials/trial-data-and-results for more information), Pfizer will provide access to individual de-identified participant data from Pfizer-sponsored global interventional clinical studies conducted for medicines, vaccines and medical devices (1) for indications that have been approved in the US and/or EU or (2) in programmes that have been terminated (i.e., development for all indications has been discontinued). Pfizer will also consider requests for the protocol, data dictionary and statistical analysis plan. Data may be requested from Pfizer trials 24 months after study completion. The de-identified participant data will be made available to researchers whose proposals meet the research criteria and other conditions, and for which an exception does not apply, via a secure portal. To gain access, data requestors must enter into a data access agreement with Pfizer.

## Abbreviations

BID: Twice daily
CFA: Confirmatory Factor Analysis
CFI: Comparative Fit Index
CI: Confidence interval
CID: Clinically Important Difference
DLQI: Dermatology Life Quality Index
PASI: Psoriasis Area and Severity Index
PGA: Physician Global Assessment
PtGA: Patient Global Assessment
