# Supplementary material for: A multi-item Physician Global Assessment scale to assess psoriasis disease severity: validation based on four phase III tofacitinib studies
Source: BMC Dermatol. 2019 Jun 7;19:8. doi: 10.1186/s12895-019-0088-2 (PMC6555979; doi:10.1186/s12895-019-0088-2)
Supplement: Supplementary file 1 — List of independent ethics committees or institutional review boards. (DOCX 47 kb) [file 12895_2019_88_MOESM1_ESM.docx]

**LIST OF INDEPENDENT ETHICS COMMITTEES OR INSTITUTIONAL REVIEW BOARDS**

| **OPT Pivotal 1 (NCT01276639)** |
| --- |
| *Canada* |
| Research Review Board Inc.  Suite 203  19 - 13085 Yonge Street  Richmond Hill, ON L4E OK2  CANADA |
| Capital Health Research Ethics Board  5790 University Avenue  Halifax, NS B3H 1V7  CANADA |
| IRB Services  Suite 300  372 Hollandview Trail  Aurora, ON L4G 0A5  CANADA |
| University of Manitoba Bannatyne  Campus Research Ethics Boards  Biomedical Research Ethics Board (BREB)  Room P126  770 Bannatyne Avenue  Winnipeg, MB R3E 0W3  CANADA |
| *Colombia* |
| Comite de Etica de la Investigacion  Riesgo de Fractura S.A.  Carrera 13 #97-25  Bogota, Cundinamarca  COLOMBIA |
| Comité de etica independiente centro de reumatologia y ortopedia  Cr. 49C No. 82-120  Barranquilla, Atlantico 0000  COLOMBIA |
| *Germany* |
| Ethik-Kommission Kiel der Christian-Albrechts-Universitaet zu Kiel  Schwanenweg 20  Kiel, 24105  GERMANY |
| *Hungary* |
| Egeszsegugyi Tudomanyos Tanacs  Klinikai Farmakologiai Etikai  Bizottsaga  Arany J. u. 6-8.  Budapest, H-1051  HUNGARY |
| *Japan* |
| Kobe University Hospital IRB  7-5-2  Kusunoki-cho Chuo-ku,  Kobe, 650-0017  JAPAN |
| Gunma University Hospital IRB  3-39-15  Showa-machi  Maebashi-shi, Gunma 371-8511  JAPAN |
| Kumamoto University Hospital IRB  1-1-1  Honjo  Kumamoto, 860-8556  JAPAN |
| JR Sapporo hospital IRB  Kita3 Higashi1  Chuo-ku  Sapporo, Hokkaido 060-0033  JAPAN |
| Japan Community Healthcare Organization Tokyo Yamate Medical Center IRB  3-22-1  Hyakunin-cho Shinjuku-ku  Tokyo, 169-0073  JAPAN |
| *Mexico* |
| Comite independiente de etica y calidad en la Investigacion soluciones Dermatologicas y medicas  S.A. DE C.V.  Paseo de los Leones No.603-A  Col.Mitras Centro  Monterrey, Nuevo Leon 64460  MEXICO |
| *Poland* |
| Komisja Bioetyczna Dolnoslaskiej  Izby Lekarskiej we Wroclawiu  ul. Matejki 6  Wroclaw, 50-333  POLAND |
| *Serbia* |
| Ethics Committee, Zvezdara  University Medical Center  Dimitrija Tucovica 161  Belgrade, 11 000  SERBIA |
| *Taiwan* |
| National Taiwan University Hospital,  Research Ethics Committee  7 Chung Shan South Road  Taipei, 100  TAIWAN |
| National Cheng Kung University Hospital,  Institutional Review Board  No. 138, Sheng-Li Road  Tainan, 704  TAIWAN |
| *Ukraine* |
| Central Committee for Ethics Issues of Ministry of Health Care of Ukraine  5, Narodnogo opolchennya Street  Kyiv, 03680  UKRAINE |
| Committee for Ethics Issues of CRI  "Clinical Dermatovenerologic Dispensary"  25, Roza Luxemburg Street  Simferopol, Crimea 95000  UKRAINE |
| Committee for Ethics Issues of SI  "Institute for Dermatology and Venerology of AMS of Ukraine"  7/9, Chernyshevska Street  Kharkiv, 61057  UKRAINE |
| Committee for Ethics Issues of Ternopil  Regional Municipal Clinical Dermatovenerologic Dispensary  39, Knyazya Ostrozkogo Street  Ternopil, 46006  UKRAINE |
| Committee for Ethics Issues of Kyiv City Dermatovenerologic Dispensary  72, Saksaganskyy Street  Kyiv, 01032  UKRAINE |
| *USA* |
| Quorum Review Institutional Review Board Incorporated  Suite 1000  1601 Fifth Avenue  Seattle, WA 98101  UNITED STATES |
| BioMedical Research Alliance of New York LLC  Institutional Review Board  1981 Marcus Avenue, Suite 210  Lake Success, NY 11042  UNITED STATES |
| Office of Research Administration  University of California, Irvine  5171 California Avenue  Irvine, CA 92697  UNITED STATES |
| Western Institutional Review Board  3535 Seventh Avenue  SW OLYMPIA, WA 98502  UNITED STATES |
| Kaiser Permanente Southern California Institutional Review Board  2nd Floor  393 East Walnut Street  Pasadena, CA 91188  UNITED STATES |
| Partners Human Research Committee  Suite 1002  116 Huntington Avenue  Boston, MA 02116  UNITED STATES |
| **OPT Pivotal 2 (NCT01309737)** |
| *Canada* |
| Research Review Board Inc.  Suite 203  19 - 13085 Yonge Street  Richmond Hill, ON L4E OK2  CANADA |
| IRB Services  Suite 300  372 Hollandview Trail  Aurora, ON L4G 0A5  CANADA |
| College of Physicians and Surgeons of Alberta - Research Ethics Review Committee  2700 Telus Plaza South  10020 - 100 Street NW  Edmonton, AB T5J 0N3  CANADA |
| Research Ethics Review Committee  Alberta Innovates-Health Solutions  1500, 10104-103 Avenue Northwest  Edmonton, AB T5J 4A7  CANADA |
| Capital Health Research Ethics Board  5790 University Avenue  Halifax, NS B3H 1V7  CANADA |
| Health Research Ethics Authority  Suite 200, Second Floor  95 Bonaventure Avenue  St. John's, NL A1B 2X5  CANADA |
| Quorum Review Institutional Review Board Inc.  Suite 1000  1601 Fifth Avenue  Seattle, WA 98101  UNITED STATES |
| *Colombia* |
| Comite de Investigaciones y Etica en Investigaciones del Hospital Pablo  Tobon Uribe  Calle 78 B No. 69-240  Medellin, Antioquia 0000  COLOMBIA |
| *Germany* |
| Ethikkommission des Fachbereichs  Medizin der Johann Wolfgang  Goethe-Universitaet Frankfurt  Haus 1  Theodor-Stern-Kai 7  Frankfurt am Main, 60590  GERMANY |
| *Hungary* |
| Egeszsegugyi Tudomanyos Tanacs  Klinikai Farmakologiai Etikai  Bizottsaga  Arany J. u. 6-8.  Budapest, H-1051  HUNGARY |
| *Mexico* |
| Comite de Etica del Instituto Dermatologico de Jalisco  Avenida Federalismo Norte 3102  Atemajac del Valle  Guadalajara, Zapopan, Jalisco 45190  MEXICO |
| Comite de Etica e Investigacion de la Unidad de Investigacion Clinica en Medicina  Unidad de Investigacion Clinica en Medicina  Avenida de la Clinica 2520 despacho 520  Colonia Sertoma  Monterrey, Nuevo Leon 64718  MEXICO |
| Comite Bioetico para la Investigacion Clinica S.C.  Puebla 422  Despacho 4  Col. Roma Sur  MEXICO, DISTRITO FEDERAL  06700  MEXICO |
| *Poland* |
| Komisja Bioetyczna przy Okregowej  Izby Lekarskiej w Lodzi  ul. Czerwona 3  Lodz, 93-005  POLAND |
| *Puerto Rico* |
| Quorum Review Institutional Review Board Inc.  Suite 1000  1601 Fifth Avenue  Seattle, WA 98101  UNITED STATES |
| *Serbia* |
| Ethics Committee Military Medical Academy  Department of Dermatology and Venereology  Crnotravska 17  Belgrade, 11000  SERBIA |
| *Taiwan* |
| Institutional Review Board of Taipei  Medical University Hospital  No.252, Wu Hsing Street  Taipei City, 110  TAIWAN |
| Chang Gung Medical Foundation,  Institutional Review Board  No. 199, Tung Hwa North Road,  Taipei, 105  TAIWAN |
| Chung Shan Medical University  Hospital Institutional Review Board  110, Section 1, Chien Kuo North Road  Taichung, Taiwan 402  TAIWAN |
| *Ukraine* |
| Central Committee for Ethics Issues of Ministry of Health Care of Ukraine  5, Narodnogo opolchennya Street  Kyiv, 03680  UKRAINE |
| Local Committee for Ethics Issues  14, Krasnodonska Street  Lugansk, 91047  UKRAINE |
| Local Committee for Ethics Issues  8, Ivana Kamysheva  Kharkiv, 61038  UKRAINE |
| Committee for Ethics Issues of Regional Municipal Dermatovenerologic Dispensary  1 Konovaltsya Street  Lviv, 79013  UKRAINE |
| Local Committee for Ethics Issues  5, Vorobyova Street  Odessa, 65006  UKRAINE |
| Committee for Ethics Issues of Kyiv  City Dermatovenerologic Dispensary  72, Saksaganskyy Street  Kyiv, 01032  UKRAINE |
| *USA* |
| Quorum Review Institutional Review Board Incorporated  Suite 1000  1601 Fifth Avenue  Seattle, WA 98101  UNITED STATES |
| WESTERN IRB  Western IRB  3535 Seventh Avenue - SW  Olympia, WA 98502-5010  UNITED STATES |
| Research Review Board Inc.  119 University Avenue East  Waterloo, ON N2J 2W1  CANADA |
| UCSD Human Research Protections Program  ECOB First Floor 9444 Medical Center Drive  La Jolla, CA 92093  UNITED STATES |
| Saint Louis University Institutional Review Board  Room C110  3556 Caroline Street  Saint Louis, MO 63104  UNITED STATES |
| Wake Forest University Health Sciences Institutional Review Board  Medical Center Boulevard  Winston-Salem, NC 27157-1042  UNITED STATES |
| Oregon Health & Science University  Institutional Review Board  3181 Southwest Sam Jackson Park Road  Portland, OR 97239  UNITED STATES |
| Committee for the Protection of Human Subjects  3rd Floor  63 South Main Street  Hanover, NH 03755  UNITED STATES |
| Penn State College of Medicine -  Penn State Milton S. Hershey  Medical Center  Human Subjects Protection Office  A115 - PO Box 855  600 Center View Drive  Hershey, PA 17033  UNITED STATES |
| Partners Human Research Committee  Suite 1002  116 Huntington Avenue  Boston, MA 02116  UNITED STATES |
| The Rockefeller University  Institutional Review Board  1230 York Avenue  New York, NY 10065  UNITED STATES |
| The University of North Carolina at Chapel Hill  Office of Human Research Ethics,  Biomedical Campus Box 7097 –  Medical School Building 52  105 Mason Farm Road  Chapel Hill, NC 27599  UNITED STATES |
| NorthShore University Health System  Institutional Review Board  2650 Ridge Avenue  Evanston, IL 60201  UNITED STATES |
| **OPT Compare (NCT01241591)** |
| *Argentina* |
| Comite de Docencia e Investigacion del Centro de Investigaciones Dermatologicas  J. E. Uriburu 1555 1° A  Ciudad Autonoma de Buenos Aires,  C1114AAP  ARGENTINA |
| Comite de Etica en Investigacion Clinica (CEIC) "Dr. Carlos A. Barclay"  Larrea 1381 - 3A  Ciudad Autуnoma de Buenos Aires,  CP1117ABK  ARGENTINA |
| Comite de Docencia e Investigacion  del IMAI Research  French 2673  Buenos Aires, C1425AWC  ARGENTINA |
| *Austria* |
| Ethikkommission der Medizinischen  Universitaet Graz  LKH-Universitaetsklinikum -  Eingangsgebaeude  Auenbruggerplatz 2, 3.OG  Graz, A-8036  AUSTRIA |
| *Belgium* |
| Commissie voor Medische Ethiek  Universitair Ziekenhuis Gent  De Pintelaan 185  Gent, 9000  BELGIUM |
| *Bosnia and Herzegovina* |
| Ethics Committee  Clinical Center University of Sarajevo  Bolnicka 25  Sarajevo, 71000  BOSNIA AND HERZEGOVINA |
| *Bulgaria* |
| Komisiya po etika pri MBAL na  Voennomeditsinska Akademia -  Sofia/Ethics Commettee at MMA  HAT-Sofia  MMA HAT-Sofia  ul. Georgi Sofiyski 3  Sofia, 1606  BULGARIA |
| Ethic Committee for Multicenter Trials  Etichna komisiya za mnogocentrovi  izpitvaniya  ul. "Damyan Gruev" 8  Sofia, 1303  BULGARIA |
| Komisiya po etika pri UMBALAlexandrovska-  Sofia/Ethics  Committee at UMHAT  Alexandrovska-Sofia  bul. Georgi Sofiyski 1  Sofia, 1431  BULGARIA |
| Komisiya po etika pri Tsentar za  kozhno-venericheski zaboliavania  EOOD  Tsentar za kozhno-venericheski  zaboliavania EOOD  ul. Georgi Izmirliev 8  Sofia, 1404  BULGARIA |
| Komisiya po etika pri UMBAL"D-r Georgi Stranski"/ Ethics Committee at MHAT "Dr. Georgi Stranski"  UMBAL"D-r Georgi Stranski"  Pleven  MHAT "Dr. Georgi Stranski" Pleven  Ul. Georgi Kochev 8A  Pleven, 5800  BULGARIA |
| Komisiya po etika pri MBALTokuda  Bolnitsa Sofia-Sofia/Ethics Committee at Hospital Tokuda Sofia  Bul. Nikola Vaptsarov 51B  Sofia, 1407  BULGARIA |
| *Chile* |
| Comite Etico Cientifico  Servicio de Salud Metropolitano  Oriente  Avenida Salvador 364  Providencia, Santiago RM 7500922  CHILE |
| Comite de Etica de Investigacion  Servicio de Salud Metropolitano  Norte  Calle San Jose # 1053  Independencia, RM 8380755  CHILE |
| Comite de Etica, Hospital Clinico  Universidad de Chile  Santos Dumont 999, 4to Piso, Sector D  Oficina 410  Independencia, Santiago, RM  CHILE |
| Comite Etico Cientifico del Servicio de Salud Vina-Quillota  Hospital Dr. Gustavo Fricke  Alvarez N° 1532,  2 Piso, Oficina 3  Vina del Mar, V Region 2570017  CHILE |
| Comite de Etica  Comite de Etica Clinica Davila  Avenida Recoleta 464  Recoleta, Santiago RM8431657  CHILE |
| *Colombia* |
| Comitй de Йtica en Investigaciones del Oriente  Calle 53 #34-20  Bucaramanga, 0000  COLOMBIA |
| Comite de Etica en Investigacion de la Clinica de la Costa  Carrera 50 No. 80-90 Segundo Piso  Barranquilla, Atlantico 0000  COLOMBIA |
| Comite de etica de la investigaciуn-  Riesgo de Fractura S.A  Carrera 13 No. 97-25  Bogota, Cundinamarca 0000  COLOMBIA |
| Comitй de Йtica Mйdica e  Investigaciуn Clнnica las Amйricas  Cra.80 Diagonal 75B No. 2A 80-140  Medellнn, Antioquia 0000  COLOMBIA |
| *Croatia* |
| Central Ethics Committee, Agency for Medicinal Products and Medical Devices  Ksaverska cesta 4  Zagreb, 10000  CROATIA |
| *Czech Republic* |
| FN Hradec Kralove  Multicentricka Eticka komise  Sokolska 581  Hradec Kralove, 50005  CZECH REPUBLIC |
| FN Plzen  Lokalni Eticka komise  Dr. E. Benese 13  Plzen-Bory, 30599  CZECH REPUBLIC |
| Eticka komise Nemocnice Ceske  Budejovice a.s.  B. Nemcove 585/54  Ceske Budejovice, 370 01  CZECH REPUBLIC |
| Eticka komise  Krajska zdravotni a.s  Masarykova nemocnice o.z.  Socialni pece 3316/12A  Usti nad Labem, 40113  CZECH REPUBLIC |
| Quintiles Laboratories Europe  The Alba Campus, Rosebank  Livingston, West Lothian EH54 7EG  UNITED KINGDOM |
| *Denmark* |
| De Videnskabsetiske Komitйer for  Region Midtjylland  Sundhedssekretariatet  Skottenborg 26  Postboks 21  Viborg, 8800  DENMARK |
| *France* |
| Comite de Protection des Personnes -  Ile-de-France IV  IV Porte 5 du carrй Historique  1 Avenue Claude Vellefaux  Paris, Cedex 10 75475  FRANCE |
| *Germany* |
| Ethikkommission an der TU Dresden  Fetscherstr. 74  Dresden, 01307  GERMANY |
| *Hong Kong* |
| Institutional Review Board of the University of Hong Kong/Hospital  Authority Hong Kong West Cluster  Rm 901  Administration Block  Queen Mary Hospital  Hong Kong,  HONG KONG |
| *Hungary* |
| Egeszsegugyi Tudomanyos Tanacs  Klinikai Farmakologiai Etikai  Bizottsaga  Arany J. u. 6-8.  Budapest, H-1051  HUNGARY |
| Intezeti Kutatasetikai Bizottsag  Korбnyi fasor 8-10.  Szeged, H-6720  HUNGARY |
| Intezeti Kutatasetikai Bizottsag  Nagyerdei kцrъt 98.  Debrecen, H-4032  HUNGARY |
| Intezeti Kutatasetikai Bizottsag  Tуszegi u 21.  Szolnok, H-5000  HUNGARY |
| Intezeti Kutatasetikai Bizottsag  Csabai Kapu 9-11  Miskolc, H-3529  HUNGARY |
| *Korea, Republic Of* |
| Medicine/Seoul National University Hospital IRB  101 Daehang-ro, Jongno-gu  Seoul, 110-744  KOREA, REPUBLIC OF |
| Severance Hospital, Yonsei University Health System,  Institutional Review Board  250 Seongsanno, Seodaemungu  Seoul, 120-752  KOREA, REPUBLIC OF |
| Samsung Medical Center  Institutional Review Board  50 Irwon-dong, Gangnam-gu  Seoul, 135-710  KOREA, REPUBLIC OF |
| *Netherlands* |
| METC Erasmus MC  Dr. Molewaterplein 50  Kamer Fd 209  Rotterdam, Zuid-Holland 3015 GE  NETHERLANDS |
| METC AMC  Meibergdreef 9  Amsterdam,  NETHERLANDS |
| METC Erasmus MC  Dr. Molewaterplein 50  Kamer Fd 209  Rotterdam, Zuid-Holland 3015 GE  NETHERLANDS |
| Erasmus Medisch Centrum  Rotterdam  Medisch Ethische Toetsings Commissie  Dr Molewaterplein 50  Rotterdam, 3015 GE  NETHERLANDS |
| METC Erasmus MC  Dr. Molewaterplein 50  Kamer Fd 209  Rotterdam, Zuid-Holland 3015 GE  NETHERLANDS |
| PT&R  Geleenbeeklaan 90  Geleen, 6166 GR  NETHERLANDS |
| *Poland* |
| Komisja Bioetyczna przy Okregowej  Izbie Lekarskiej w Krakowie  ul. Krupnicza 11a  Krakow, 31-123  POLAND |
| *Russian Federation* |
| Ethics Committee of Moscow State Medical Stomatological University  Stroenie 1, 20 ulitsa Delegatskaya  Moscow, 127473  RUSSIAN FEDERATION |
| Ethics Committee of State Research Center of Dermatovenerology  Stroenie 6, 3 ulitsa Korolenko  Moscow, 107076  RUSSIAN FEDERATION |
| Independent Ethics Committee at the Military Medical Academy S.M.  Kirov  6 ulitsa Akademika Lebedeva  Saint-Petersburg, 194044  RUSSIAN FEDERATION |
| Ethics Committee of Saratov State Medical University V. I.  Razumovsky  112 ulitsa Bolshaya Kazachiya  Saratov, 410012  RUSSIAN FEDERATION |
| Ethics Committee of Ryazan regional clinical dermatovenerologic dispensary  9 ulitsa Sportivnaya  Ryazan, 390046  RUSSIAN FEDERATION |
| Ethics Committee at the Rostov-on-Don Regional Dermatovenerologic Dispensary  70 ulitsa Baumana  Rostov-on-Don, 344007  RUSSIAN FEDERATION |
| Local Ethics Committee  North-Western State Medical University I.I. Mechnikov  41 ulitsa Kirochnaya  Saint-Petersburg, 191015  RUSSIAN FEDERATION |
| Independent Ethics Committee at Smolensk State Medical Academy  28 ulitsa Krupskoy  Smolensk, 214019  RUSSIAN FEDERATION |
| Ethics Committee at Clinical Hospital of Emergency Care N.V.  Soloviev  11 ulitsa Zagorodnyj sad  Yaroslavl, 150003  RUSSIAN FEDERATION |
| *Singapore* |
| SingHealth Centralized Institutional Review Board  Singapore Health Services Pte Ltd  Blk A, 7 Hospital Drive,  SingHealth Research Facilities, #03-01  Singapore, Singapore 169611  SINGAPORE |
| Domain Specific Review Board (DSRB)  National Healthcare Group  Research & Development Office  6 Commonwealth Lane, Level 6  GMTI Building  Singapore, Singapore 149547  SINGAPORE |
| *Slovakia* |
| Eticka komisia  Fakultna nemocnica s poliklinikou  F.D.Roosevelta Banska Bystrica  Namestie L.Svobodu 1  Banska Bystrica, 975 17  SLOVAKIA |
| Eticka komisia  Narodny ustav reumatickych chorob  Nabrezie I.Krasku 4  Piestany, 921 12  SLOVAKIA |
| *Spain* |
| Hospital General de Alicante  Comite Etico de Investigacion Clinica  C/ Maestro Alonso, 109  Alicante, Alicante 03010  SPAIN |
| Hospital Puerta de Hierro Majadahonda  Ethics Committee Of Clinical Investigation  Secretaria Tecnica  C\ Joaquin Rodrigo, 2 - Planta 1Є -  Pasillo Unidades Administrativas  (PEINES 6-7)  Majadahonda, Madrid 28222  SPAIN |
| Consorcio Hospital General Universitario de Valencia  Comite Etico de Investigacion Clinica  Pabellon B - Acceso B-3, 4Є Planta  Avda. Tres Cruces, s/n  Valencia, Valencia 46014  SPAIN |
| Hospital Universitario de la Princesa  Comite Etico de Investigacion Clinica  C/ Diego de Leon 62  Madrid, 28006  SPAIN |
| Hospital 12 de Octubre Instituto de Investigacion  Hospital 12 de Octubre (i+12)  Area de Gestion de Proyectos -  Unidad Administrativa CEIC  Centro de Actividades Ambulatorias,  Bloque D - Planta 6Є  Avda de Cordoba s/n  Madrid, Madrid 28041  SPAIN |
| *Sweden* |
| Regionala etikprovningsnamnden  i Stockholm  Box 289  Stockholm, 17 177  SWEDEN |
| *Switzerland* |
|  |
| *Turkey* |
| Ilac Klinik Arastirmalar Etik  Danisma Kurulu  Sogutozu Mahallesi 2176. Sokak  No:5  Cankaya, Ankara 06520  TURKEY |
| Istanbul Universitesi Istanbul Tip  Fakultesi Klinik Arastirmalar Etik  Kurulu  Istanbul, Capa 34390  TURKEY |
| Istanbul University Faculty of Medicine Ethics Committee  Capa, Istanbul  TURKEY |
| T.R. Ministry of Health General  Directorate of Pharmaceuticals and Pharmacy  Sogutozu Mahallesi 2176. Sokak  No:5  Cankaya, Ankara 06520  TURKEY |
| *United Kingdom* |
| NRES Committee London-Surrey Borders Charing Cross Hospital, Research Ethics Committee (REC) Centre  Charing Cross  Room 12, 4th Floor West  Fulham Palace Road  London, W6 8RF  UNITED KINGDOM |
| R&D Office  Leicester General Hospital  Sharon Turner  Leicester, LE5 4PW  UNITED KINGDOM |
| **OPT Retreatment (NCT01186744)** |
| *Argentina* |
| Comite de Docencia e Investigacion  del IMAI Research  French 2673  Buenos Aires, C1425AWC  ARGENTINA |
| Comite de Etica en Investigacion Clinica (CEIC) "Dr. Carlos A. Barclay"  Larrea 1381 Piso 3 "A"  Buenos Aires, C1117ABK  ARGENTINA |
| Comite de Docencia e Investigacion del Centro de Investigaciones Dermatologicas  J. E. Uriburu 1555 1° A  Ciudad Autonoma de Buenos Aires,  C1114AAP  ARGENTINA |
| *Australia* |
| Bellbery Human Research Ethics Committee  229 Greenhill Road  Dulwich, SA 5065  AUSTRALIA |
| *Brazil* |
| Comitê de Ética em Pesquisa da Faculdade de Medicina da Universidade de São Paulo -  FMUSP/SP  Instituto Oscar Freire da Faculdade de Medicina da Universidade de São Paulo  Av. Dr. Arnaldo, 455 - 1 andar  São Paulo, São Paulo 01246-903  BRAZIL |
| Comitê de Ética em Pesquisa da Santa Casa de Misericórdia do Rio de Janeiro  Rua Santa Luzia, 206  Rio de Janeiro, RJ 20020-022  BRAZIL |
| *Bulgaria* |
| Ethic Committee for Multicenter Trials  Etichna komisiya za mnogocentrovi izpitvaniya ul. "Damyan Gruev" 8  Sofia, 1303  BULGARIA |
| Komisiya po etika pri MBAL na Voennomeditsinska Akademia -  Sofia/Ethics Committee at MMA HAT-Sofia  MMA HAT-Sofia  ul. Georgi Sofiyski 3  Sofia, 1606  BULGARIA |
| Komisiya po etika pri UMBALAlexandrovska-  Sofia/Ethics Committee at UMHAT  Alexandrovska-Sofia  bul. Georgi Sofiyski 1  Sofia, 1431  BULGARIA |
| Komisiya po etika pri ODKVBZSSofia/  Ethics Committee at DDSVDWS-Sofia  ul. Georgi Izmirliev 8  Sofia, 1404  BULGARIA |
| Komisiya po etika pri Tsentar za  kozhno-venericheski zaboliavania  EOOD  Tsentar za kozhno-venericheski  zaboliavania EOOD  ul. Georgi Izmirliev 8  Sofia, 1404  BULGARIA |
| Etychna Comissia Za Universitetska  Mnogoprofilna B  91, General Vladimir Vazov Str.  Pleven, 5800  BULGARIA |
| Komisiya po etika pri UMBAL"D-r  Georgi Stranski"/ Ethics Committee  at MHAT "Dr. Georgi Stranski"  UMBAL"D-r Georgi Stranski"  Pleven  MHAT "Dr. Georgi Stranski" Pleven  Ul. Georgi Kochev 8A  Pleven, 5800  BULGARIA |
| Komisiya po etika pri MBALTokuda  Bolnitsa Sofia-Sofia/Ethics Committee at Hospital Tokuda Sofia  Bul. Nikola Vaptsarov 51B  Sofia, 1407  BULGARIA |
| *Canada* |
| IRB Services  Suite 300  372 Hollandview Trail  Aurora, ON L4G 0A5  CANADA |
| UBC Clinical Research Ethics Office  Room 210, Research Pavilion  828 West 10th Avenue  Vancouver, BC V5Z 1L8  CANADA |
| *Denmark* |
| De Videnskabsetiske Komitéer for  Region Midtjylland  Sundhedssekretariatet  Skottenborg 26  Postboks 21  Viborg, 8800  DENMARK |
| *Finland* |
| Pirkanmaa Hospital District Ethics Committee  Kirsi Kohonen  PL 2000 (Biokatu 12)  Tampere, 33521  FINLAND |
| *Greece* |
| National Ethics Committee  Ministry of Health & Social Solidarity  284, Mesogeion Av.  Cholargos  Athens, 15562  GREECE |
| *Netherlands* |
| IRB Nijmegen  Heilige Stoel 44-02  Wijchen, 6601 VZ  NETHERLANDS |
| *Slovakia* |
| Eticka komisia  Fakultna nemocnica Trnava  A. Zarnova 11  Trnava, 917 75  SLOVAKIA |
| Eticka komisia  Univerzitna nemocnica Bratislava a  LFUK, Nemocnica Stare mesto  Mickiewiczova 13  Bratislava, 813 69  SLOVAKIA |
| *United Kingdom* |
| NRES Committee London - Central  Level 7, Maternity Block  Northwick Park Hospital  Watford Road  Harrow, HA1 3UJ  UNITED KINGDOM |
| R&D Department, Whipps Cross  University Hospital  Whipps Cross Road  Leytonstone, E11 1NR  UNITED KINGDOM |
| R&D Governance Officer  (Approvals), Salford Royal NHS  Foundation Trust  Research & Development  Summerfield House  554 Eccles New Road  Salford, M5 5AP  UNITED KINGDOM |
| *USA* |
| Quorum Review Institutional Review Board Incorporated  Suite 1000  1601 Fifth Avenue  Seattle, WA 98101  UNITED STATES |
| The Committee on the Protection of Human Subjects  Rhode Island Hospital  Aldrich 3  593 Eddy Street  Providence, RI 02903  UNITED STATES |
| University of California San Francisco Committee on Human Research  Office of Research  Suite 315 - Box 0692  3333 California Street  San Francisco, CA 94118  UNITED STATES |
| University Hospitals Case Medical Center Institutional Review Board for Human Investigation  The Center for Clinical Research  11100 Euclid Avenue  Cleveland, OH 44106  UNITED STATES |
| Duke University Health System  Institutional Review Board  Hock Plaza  Suite 405  2424 Erwin Road  Durham, NC 27705  UNITED STATES |
